# Dysfunctional Presynaptic M_2_ Receptors in the Presence of Chronically High Acetylcholine Levels: Data from the PRiMA Knockout Mouse

**DOI:** 10.1371/journal.pone.0141136

**Published:** 2015-10-27

**Authors:** Franziska Mohr, Eric Krejci, Martina Zimmermann, Jochen Klein

**Affiliations:** 1 Department of Pharmacology, School of Pharmacy, Goethe University, Frankfurt am Main, Germany; 2 Centre d’Etude de la Sensorimotricité, Université Paris Descartes, CNRS UMR 8194, Paris, France; 3 Centre for the Humanities and Health, Department of English, King´s College, London, United Kingdom; Weizmann Institute of Science, ISRAEL

## Abstract

The muscarinic M_2_ receptor (M2R) acts as a negative feedback regulator in central cholinergic systems. Activation of the M_2_ receptor limits acetylcholine (ACh) release, especially when ACh levels are increased because acetylcholinesterase (AChE) activity is acutely inhibited. Chronically high ACh levels in the extracellular space, however, were reported to down-regulate M2R to various degrees. In the present study, we used the PRiMA knockout mouse which develops severely reduced AChE activity postnatally to investigate ACh release, and we used microdialysis to investigate whether the function of M2R to reduce ACh release *in vivo* was impaired in adult PRiMA knockout mice. We first show that striatal and hippocampal ACh levels, while strongly increased, still respond to AChE inhibitors. Infusion or injection of oxotremorine, a muscarinic M_2_ agonist, reduced ACh levels in wild-type mice but did not significantly affect ACh levels in PRiMA knockout mice or in wild-type mice in which ACh levels were artificially increased by infusion of neostigmine. Scopolamine, a muscarinic antagonist, increased ACh levels in wild-type mice receiving neostigmine, but not in wild-type mice or in PRiMA knockout mice. These results demonstrate that M2R are dysfunctional and do not affect ACh levels in PRiMA knockout mice, likely because of down-regulation and/or loss of receptor-effector coupling. Remarkably, this loss of function does not affect cognitive functions in PRiMA knockout mice. Our results are discussed in the context of AChE inhibitor therapy as used in dementia.

## Introduction

G protein-coupled receptors (GPCR) are workhorses for neurotransmitter actions, mediating pre- as well as postsynaptic effects. In the central cholinergic system, muscarinic M_1_ receptors are the main receptor subtype for postsynaptic actions, e.g. in hippocampus and cortex, whereas M_2_/M_4_-type receptors are often located pre-synaptically, where they limit release of acetylcholine (ACh) under conditions of high neuronal activity [1,2]. When ACh levels rise rapidly, e.g. after administration of an acetylcholinesterase (AChE) inhibitor, presynaptic muscarinic receptors M_2_ (M2R) limit ACh release, but this limitation can be overcome by concomitant application of muscarinic antagonists [3,4]. This phenomenon has given rise to the development of muscarinic M_2_ receptor antagonists as potential drugs for the treatment of cholinergic dysfunction [5,6].

An interesting phenomenon in this respect is the observation that M2R are down-regulated when synaptic ACh levels remain high for extended periods of time. This is the case in transgenic mouse models with reduced AChE activity. In AChE knockout mice, for instance, M_2_ receptors are strongly down-regulated [7,8] as a consequence of extremely high ACh levels [9]. Behavioral consequences, however, cannot be reliably investigated in these mice due to a severe phenotype [10]. In a different model, the PRiMA knockout mouse, AChE activity in the brain is strongly reduced but not absent [11]. These mice are phenotypically normal, and motor function shows only minor and difficult-to-detect changes, although ACh levels in the brain are extremely high in these mice as well [12]. Muscarinic receptors, especially the M_2_ subtype, are down-regulated by 20–60% depending on the brain region investigated [12], and this down-regulation occurs in parallel with the development of the central cholinergic fibres post-natally [13].

In the present work, we have tested the functionality of presynaptic M_2_ receptors by testing the effects of a muscarinic agonist and an antagonist on the release of ACh. ACh release was determined by microdialysis *in vivo*. The rationale for this study was the consideration that a reduction of muscarinic receptors does not automatically imply that they stop being functional. GPCR can be down-regulated by endocytosis and degraded, but they can also be recycled from an intracellular pool of receptors and remain active. These pathways have been investigated in some detail in the case of adrenergic and opioid receptors [14,15]. Microscopic investigations of receptor numbers distinguish between membrane-bound and intracellular receptors, but data so obtained do not easily answer quantitative questions. On the other hand, ligand-binding assays give quantitative information but do not distinguish active from inactive receptors. None of these assays can investigate receptor-effector coupling *in vivo*. Therefore, in the present study, we investigated the functionality of M2R in an assay measuring ACh release *in vivo*, using microdialysis. We report that, in our assay with ACh as an endpoint, presynaptic muscarinic receptors become completely inactive in PRiMA knockout mice. This finding has consequences for the chronic treatment of dementia patients with inhibitors of AChE.

## Methods

### Ethics statement

All experiments were conducted in accordance with the guidelines set out by the responsible government agency (Regierungspräsidium Darmstadt, Germany) and our study was approved by the Regierungspräsidium Darmstadt under protocol F8/23. All experiments were performed so as to minimize animal suffering.

### Animals

The PRiMA knockout (KO) mice were created as previously described (Dobbertin *et al*., 2009) and are maintained on a mixed B6D2 genetic background. Mice were kept under standardised light/dark (12/12 h), temperature (22°C) and humidity (45%) conditions, with rodent diet and water available *ad libitum*. All experiments were performed using adult (3–4 months old) female mice from different litters.

### Microdialysis

Neurotransmitter release was assessed in murine hippocampus or striatum. On day 1, mice were anesthetised with isoflurane (4% induction, 1.5% maintenance) in synthetic air and placed in a stereotaxic frame. I-shaped concentric dialysis probes with an exchange length of 2 mm were constructed as previously described [16] and implanted into the right striatum using the following coordinates (from bregma): AP: 0.5 mm, L: –2.2 mm, and DV: –3.8 mm; for hippocampal measurements, the following coordinates (from bregma) were used: AP: -2.7 mm, L: –3.0 mm, and DV: –3.75 mm. Multilink® Automix resin (Ivoclar Vivadent AG, Schaan, Liechtenstein) was used to fix the probe onto the skull. Animals were allowed to recover for 24 h. Experiments were carried out in freely moving animals on the following two days. Microdialysis probes were perfused with artificial cerebrospinal fluid (aCSF: 147 mM NaCl, 2.7 mM KCl, 1.2 mM MgCl_2_ and 1.2 mM CaCl_2_; all chemicals from VWR, Darmstadt, Germany) at a perfusion rate of 1 μl/min. Dialysate was sampled at intervals of 15 min in order to obtain baseline values for a 60 min time span. Subsequently, the dialysis fluid was switched to aCSF containing appropriate concentrations of drug for local administration (1 μM BW284c51, bambuterol, or scopolamine; 100 μM oxotremorine; all from Sigma-Aldrich, Munich, Germany). After 90 or 120 min, the perfusion fluid was switched back to drug-free aCSF, and mice were further monitored for a period of at least 1 h. Alternatively, mice received drugs by systemic (i.p.) administration (1 μmol/kg neostigmine or physostigmine; 0.5 mg/kg oxotremorinee or scopolamine), and microdialysis was continued for four hours. Samples were stored on ice immediately after collection with a fraction collector (Biorad, Munich, Germany), and frozen to –20°C until analysis was carried out. Between dialysis days, animals were kept in their home cages with food and water available *ad libitum*. On day 4, the animals were sacrificed by decapitation in isoflurane anesthesia and their brains extracted. Following the preparation of 1 mm coronal sections, the correct microdialysis probe location (in striatal or hippocampal tissue) was verified.

### Measurement of acetylcholine and acetylcholinesterase

Acetylcholine (ACh) and choline (Ch) in dialysates were determined by microbore HPLC-ECD, using the Eicom HTEC-500 system (Kyoto, Japan) equipped with low-speed pump, pre- and separation column, enzyme reactor carrying immobilised AChE and choline oxidase, and electrochemical detector with a platinum electrode operating at 500 mV. The mobile phase consisted of KHCO_3_ 50 mmol/L (Merck, Darmstadt, Germany), EDTA-2Na 134.3 μmol/L (BDH, Poole, UK) and sodium decane-1-sulfonate 1.64 mmol/L (Alfa Aesar, Karlsruhe, Germany) in RotisolV HPLC gradient grade water (Sigma Aldrich, Munich, Germany), brought to pH 8.4. The flow rate was 150 μL/min. At an injection volume of 5 μL, the detection limit of this system was 1–2 fmol/injection. Intra-assay and inter-assay coefficients of variability have previously been described [16]. Data acquisition was performed using the EPC-500 PowerChrom® software.

Acetylcholinesterase activity was measured in brain homogenates using the Ellman assay with minor modifications as described [17].

### Behavior tests

Mice were allowed to adapt to an altered day/night cycle with the light phase being changed to the period from 3 a.m. to 3 p.m. over 7 days prior to testing. Experiments were carried out in a dark (red light) and quiet environment starting at 4 p.m. Tests were performed as previously described [16]. Briefly, to test object recognition, the mouse was placed in a freshly cleaned open field box (45 x 30 cm) containing two similar objects (cylinders), and explorative contacts between mouse and objects were counted for 5 min. 24 h later, the same mouse was placed in the open field box again, but now exposed to one of the familiar cylinders and one unfamiliar cube, with explorative contacts being counted, again, for 5 min. To test social recognition, the mouse was placed in a freshly cleaned open field box (75 x 43 cm) containing two small round cages (7.5 cm diameter). During session 1, an unfamiliar mouse was placed in one of the small cages. For 5 min, contacts between the freely moving mouse and the empty cage were counted as compared to contacts with the cage containing the unfamiliar mouse (including contacts with that unfamiliar mouse). After 10 min, the experiment was repeated. Now, the originally empty small cage contained a second unfamiliar mouse, while the other small cage contained the originally unfamiliar (now already known) mouse. Explorative contacts were counted, again, for 5 min. All mice were female and taken from different litters. To test passive avoidance, a closed dark box (30 x 30 x 30 cm) connected to an open air platform (5 x 4 cm; 20 cm above ground; illuminated by a bright light), from where the mouse can enter the box through a little hole (3 x 3 cm), was used. The floor of the box was fitted with a metal mesh connected to a power source. The training session consisted of placing the mouse on the platform and measuring the time the mouse takes to enter the box; once the mouse had entered the box, an electric shock (1 mA) was applied. 24 h later, the experiment was repeated, but this time, no electric shock was applied, once the mouse had entered the box; time was stopped after 3 min or with the mouse entering the box [18].

### Data analysis and statistical evaluation

Basal ACh levels in WT and PRiMA KO mice were compared by Student’s t-test, while ACh time course data as obtained by microdialysis was analysed using a two-way analysis of variance (ANOVA) of repeated measures and Bonferroni’s post hoc test (analysis was carried out using GraphPad Prism®). Non-parametric data (passive avoidance procedure) was analysed using the Wilcoxon test suitable for the comparison of paired, non-Gaussian distributed data. Significance of data was assumed when statements could be made with 95% confidence. The graphical presentation of data refers to absolute values and is given as mean values ± standard error of the mean (SEM) or ± standard deviation (SD), with the number of experiments being indicated in figure legends.

## Results

### Baseline levels of ACh and AChE and effects of AChE inhibitors

In agreement with previous data, AChE activity in brain homogenates of PRiMA KO mice was only 7.7% of wild-type controls (0.09 ± 0.02 U/mg vs. 1.11 ± 0.34 U/mg, mean ± S.D., N = 6 each). As a consequence, in a large animal cohort, the extracellular concentration of ACh in the striatum of PRiMA KO mice was 400-fold higher than in wild-type mice (281 ± 154 nmol/l vs. 0.71 ± 0.50 nmol/l, N = 25; these data were not corrected for *in vitro*-recovery of the probes which was 11.8% for ACh). In the hippocampus, ACh levels in PRiMA KO mice were increased 40-fold, from 0.98 ± 0.42 nmol/l to 41.5 ± 8.96 nmol/l (N = 7). Notably, these ACh levels were assessed without adding an AChE inhibitors to the perfusion fluid.

Extracellular concentrations of choline were lower in PRiMA KO mice as compared to WT mice: in PRiMA KO mice, choline levels were 1591 ± 839 nmol/l in the striatum and 1057 ± 425 nmol/l in the hippocampus, whereas the values in WT mice were 1964 ± 728 nmol/l and 1375 ± 388 nmol/l, respectively (not corrected for *in vitro*-recovery which was 11.5% for choline) (data not illustrated).

**Fig 1:**
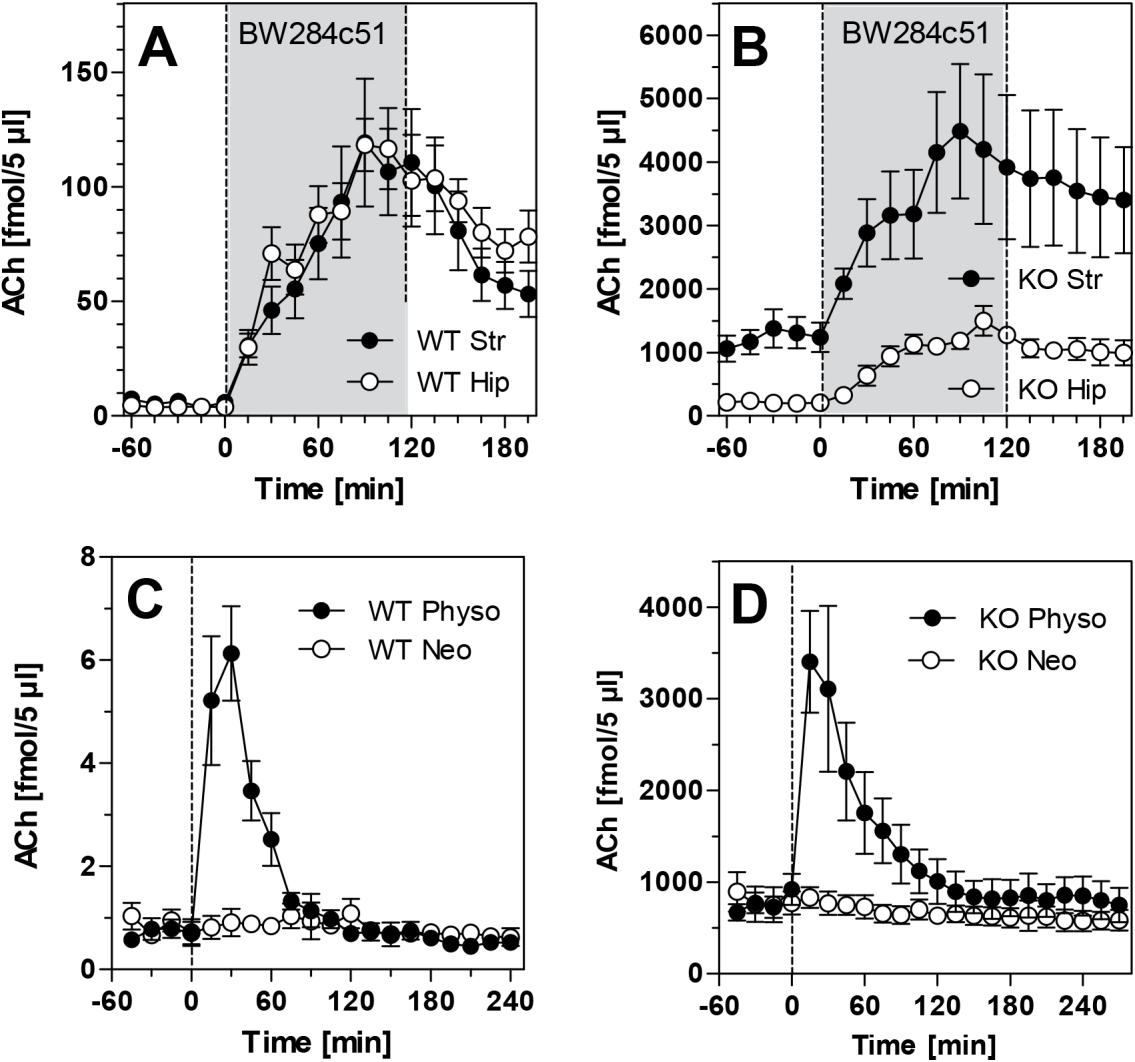
(A, B) Efflux of acetylcholine (ACh) from murine hippocampus during local perfusion with BW284c51, a specific AChE inhibitor. BW284c51was administered directly through the microdialysis probe by infusing it with the aCSF fluid for 120 min starting at time point zero (grey area). Data is presented as means ± SEM (N = 7) and given as absolute values, not corrected for *in-vitro* recovery. (C, D) Efflux of acetylcholine (ACh) from murine striatum following systemic administration of AChE inhibitors. Administration of neostigmine (1 μmol/kg i.p.) or physostigmine (1 μmol/kg i.p.) in (C) wild-type mice and (D) PRiMA knockout mice at time point zero. Data is presented as means ± SEM of 4–7 experiments and given as absolute values, not corrected for *in-vitro* recovery. Raw data are given in the S1 File.

Fig 1 illustrates that these ACh levels, albeit very high in PRiMA KO mice, were still regulated by AChE activity. When the highly selective AChE inhibitor BW284c51 (IC_50_ for AChE: 18.8 nmol/l) was infused locally into the striatum or hippocampus of WT mice at a concentration of 1 μM, ACh levels increased more than 20-fold (from 0.71 ± 0.50 to 23.9 ± 4.2 nmol/l in striatum and from 0.98 ± 0.42 to 23.6 ± 1.7 nmol/l in hippocampus) within 120 min of infusion onset (Fig 1A). Systemic administration of the AChE inhibitor physostigmine (1 μmol/kg i.p.) to WT mice also increased striatal levels of ACh approx. tenfold, to 1.63 ± 0.14 nmol/l (Fig 1C). In contrast, administration of the AChE inhibitor neostigmine, which does not cross the blood-brain barrier, was ineffective in all mice (Fig 1C and 1D). In PRiMA KO mice, BW284c51 also caused several-fold increases of extracellular ACh levels to 897 ± 34 nmol/l in striatum and to 236 ± 19 nmol/l in hippocampus (Fig 1B). Physostigmine increased striatal ACh levels to 622 ± 180 nmol/l (Fig 1D). In parallel experiments in striatum and hippocampus, bambuterol, one of the most specific inhibitors of butyrylcholinesterase (BChE), was locally infused through the microdialysis at 1 μM, but did not affect ACh levels in either mouse type (data not shown).

### Functionality of muscarinic M_2_ receptors

**Fig 2:**
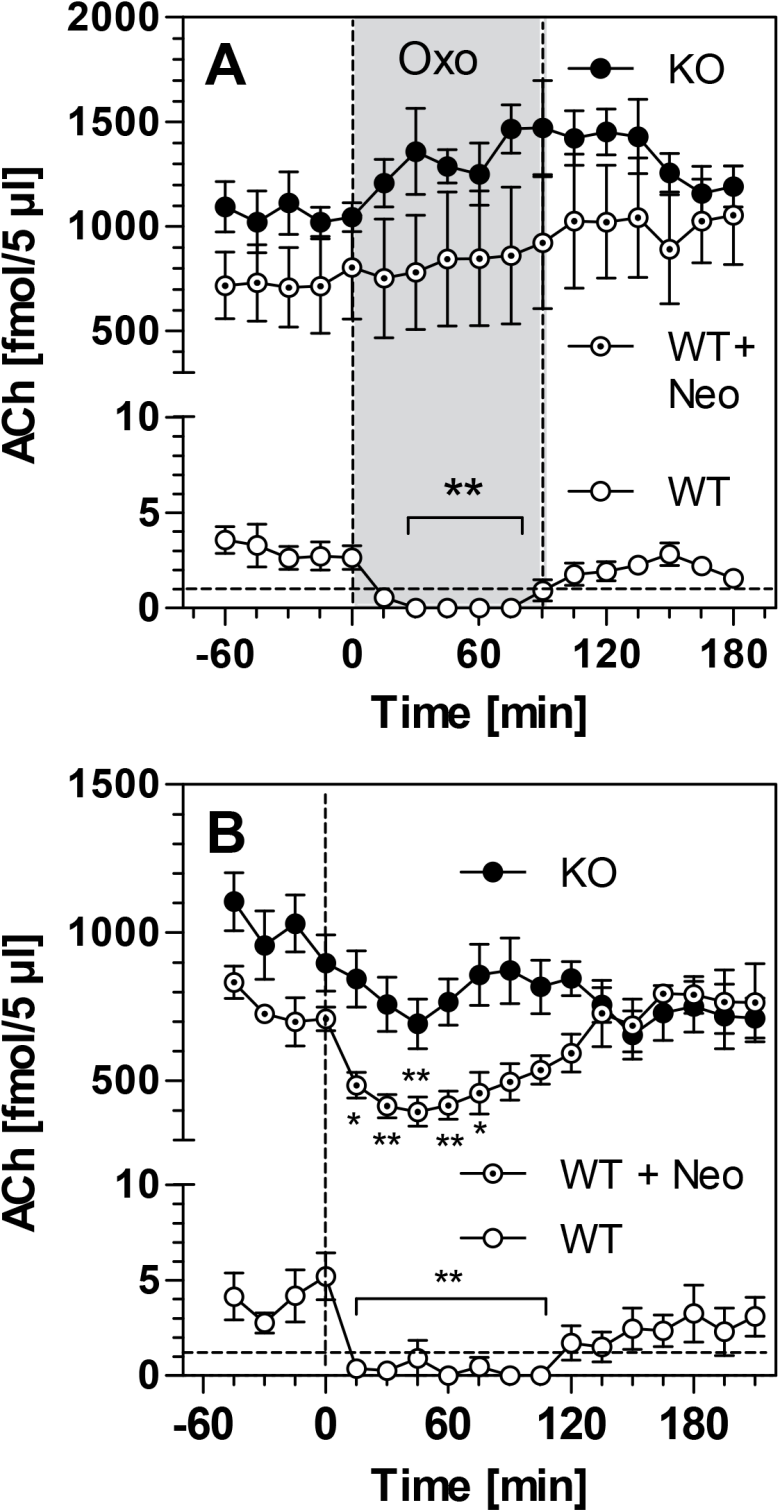
Acetylcholine (ACh) levels in murine striatum following administration of the M2 agonist oxotremorine. Data is presented for WT mice, WT+neo mice (group of WT mice additionally receiving 1 μM neostigmine through the microdialysis probe to increase ACh levels) and PRiMA KO mice. Data is presented as means ± SEM of 4–6 experiments and given as absolute values, not corrected for *in-vitro* recovery. **(A)** ACh levels measured during oxotremorinee infusion (OXO, 100 μM) indicated by grey area. **(B)** ACh levels measured after i.p. injection of oxotremorine (0.5 mg/kg) at time-point zero (dashed line). Statistical significance was calculated by One-way ANOVA with Dunnett´s Multiple Comparison Test, using time point "0 min" as reference. *, p<0.05; **, p<0.01 vs. time zero. Raw data are given in S1 File.

To test the response of presynaptic M_2_ receptors to muscarinic agonists, we administered oxotremorine both locally and systemically. Local infusion of oxotremorine into mouse striatum caused a drop of striatal ACh levels below the detection limit in WT mice (Fig 2A). However, oxotremorine infusion did not affect the high ACh levels found in PRiMA KO mice (Fig 2A). To mimic the situation of such high ACh levels in WT mice, we performed an additional experiment. We first infused neostigmine (1 μM) to the perfusion fluid of WT mice and then added oxotremorine. Under this condition, oxotremorine was no longer able to affect ACh levels in WT mice (Fig 2A). Similar but distinct effects were observed after systemic, i.p. injection of oxotremorine (0.5 mg/kg) (Fig 2B). Systemic oxotremorine strongly reduced ACh levels in WT mice below detection limit. In PRiMA KO mice, systemic oxotremorine led to a moderate decrease of striatal ACh levels from 898 ± 94 fmol/5 μL to 709 ± 87 fmol/5 μL (p>0.2, paired t-test). Similarly, in wild-type mice infused with 1 μM neostigmine, oxotremorine reduced the average ACh level of 710 ± 79 fmol/5 μL to 396 ± 97 fmol/5 μL (p<0.05, paired t-test) (Fig 2B).

**Fig 3:**
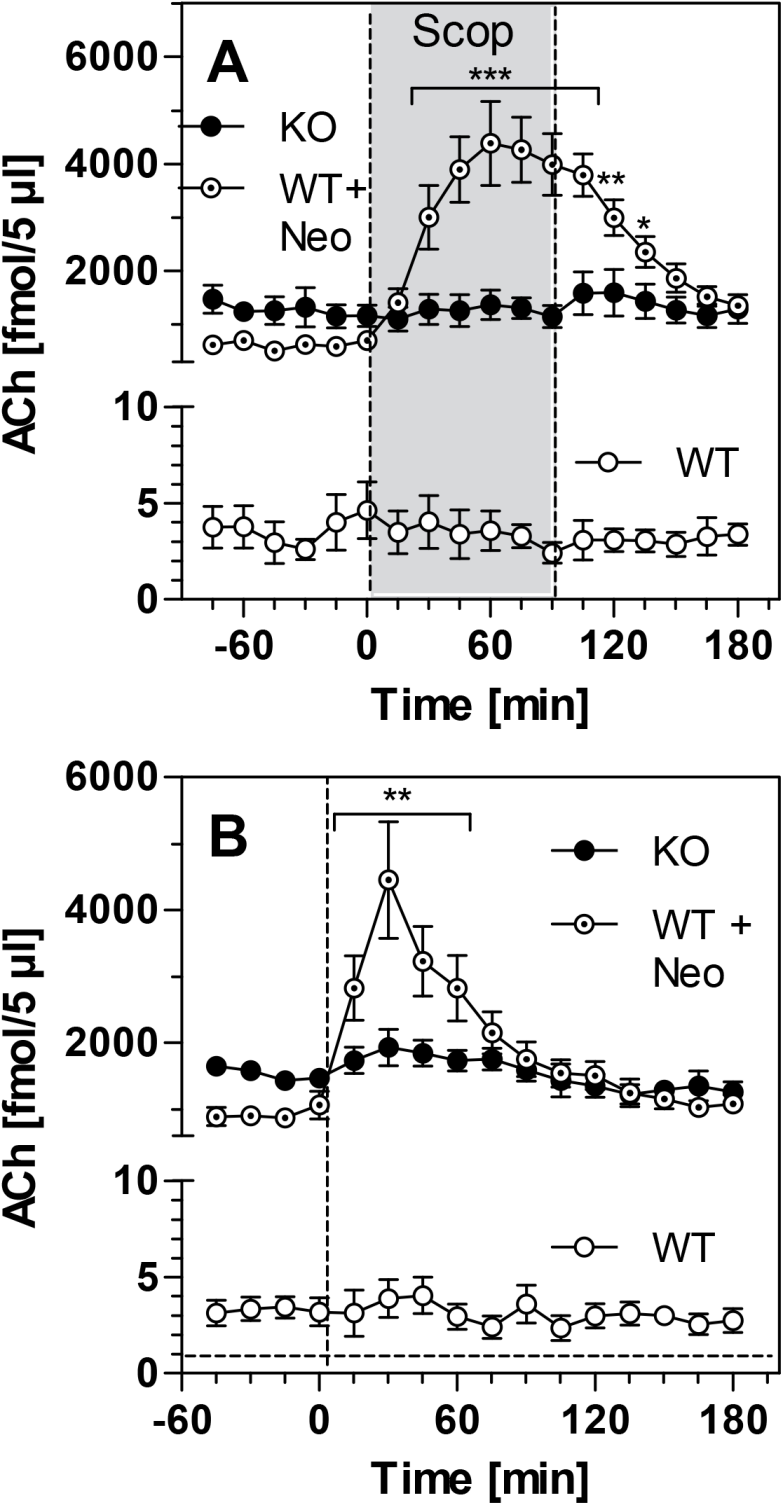
Acetylcholine (ACh) levels in murine striatum following administration of the M2 antagonist scopolamine. Data is presented for WT mice, WT+Neo mice (group of WT mice additionally receiving 1 μM neostigmine through the microdialysis probe to raise ACh levels) and PRiMA KO mice. Data is presented as means ± SEM of 5 experiments and given as absolute values, not corrected for *in-vitro* recovery. **(A)** ACh levels measured during scopolamine infusion (SCOP, 1 μM) indicated by grey area. **(B)** ACh levels measured after i.p. injection of scopolamine (0.5 mg/kg) at time-point zero (dashed line). Statistical significance was calculated by One-way ANOVA with Dunnett´s Multiple Comparison Test, using time point "0 min" as reference. *, p<0.05; **, p<0.01; ***, p<0.001 vs. time zero. Raw data are given in S1 File.

To test the response of presynaptic M_2_ receptors towards muscarinic antagonists, we administered scopolamine both locally and systemically. Local infusion of scopolamine into mouse striatum increased striatal ACh levels in WT mice, but only when they were artificially enhanced by the presence of neostigmine (Fig 3A); this effect was significant (ACh at time zero: 701 ± 117 fmol/5 μL; at 60 min: 4383 ± 788 fmol/5 μL; p<0.01). Scopolamine infusion did not affect the high ACh levels found in PRiMA KO mice (Fig 3A). Similar effects were observed after systemic, i.p. injection of scopolamine (0.5 mg/kg) (Fig 3B). Systemic scopolamine strongly increased ACh levels in WT mice, but again only in the presence of neostigmine (ACh at time zero: 1067 ± 205 fmol/5 μL; at 30 min: 4454 ± 879 fmol/5 μL; p<0.01). In PRiMA KO mice, in contrast, systemic scopolamine was ineffective (Fig 3B).

### Cognitive function in PRiMA KO mice

**Fig 4:**
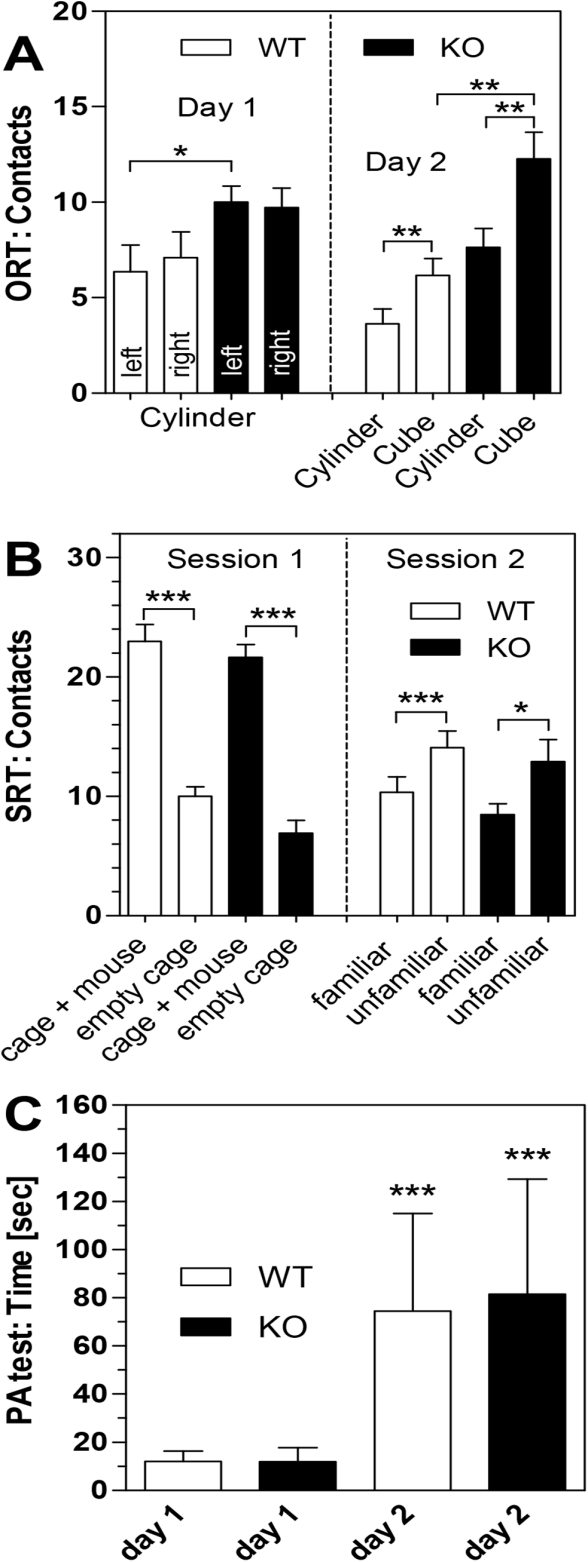
Cognitive function in WT and PRiMA KO mice. (**A**) Object recognition test. (**B**) Social recognition test. (**C**) Passive avoidance test. Data are given as means ± S.E.M of 11 experiments. For details of test procedures, see the Materials and Methods section. Statistical significance was calculated by paired t-test in (A) and (B) and by paired Wilcoxon rank test for non-parametric data in (C) (*, p<0.05; **, p<0.01; ***, p<0.001). Raw data are given in the S1 File.

In order to assess whether high levels of ACh and dysfunctional M_2_ receptors in the PRiMA KO mice affects cognitive behavior, three different tests were performed, all of which centred on memory consolidation. As shown in Fig 4A, in the object recognition test (ORT), both PRiMA KO and WT mice responded with curiosity to a new object (cube) on day 2 of the test, making significantly less contact with the known cylinder (contacts on day 2: WT: 6.18 ± 0.88 for cube vs. 3.63 ± 0.78 for cylinder; PRiMA KO: 12.27 ± 1.34 for cube vs. 7.64 ± 0.98 for cylinder; both N = 11 and p<0.01). Additionally, we observed that PRiMA KO mice showed more curiosity overall as manifested in total contacts (WT day 1: 13.5 ± 2.65 contacts vs. PRiMA KO day 1: 19.7 ± 1.59 contacts; p = 0.055; WT day 2: 9.8 ± 1.55 contacts vs. PRiMA KO day 2: 19.0 ± 1.98 contacts; p < 0.01).

The social recognition test revealed great similarities between the PRiMA KO and the WT mouse behavioral phenotype. Both strains were significantly more interested in a mouse than an empty cage during the first session (Fig 4B), while in the second session, an unfamiliar mouse sparked more interest than the already known mouse. There were no statistically significant differences between the two mouse strains. Finally, passive avoidance behavior revealed that both strains showed significant learning behavior because they stayed for much longer on the lighted platform on the second day of experimentation, after having been exposed to an electric shock the previous day (Fig 4C). Again, both mouse strains performed equally well.

## Discussion

The present study of adaptive processes in the central cholinergic system was motivated by the use of AChE inhibitors in human disease, primarily for the treatment of dementias such as Alzheimer´s disease (AD) [19,20]. While the effects of acute treatment with AChE inhibitors are well known from animal studies, animal models for chronic and stable inhibition of AChE are more difficult to find. The AChE KO mouse is a model of permanent AChE deficiency, but although this mouse is viable [10], it is very sick and hardly useful for translational purposes. Nevertheless, some adaptive processes such as down-regulation of muscarinic receptors have been described in these mice (see Introduction). The heterozygous AChE +/- mouse has 40–50% residual AChE activity, is phenotypically healthy and behaviorally normal [16]. However, in these mice, ACh levels are only increased by about two-fold, a situation in which an adaptation to chronically increased ACh levels may not necessarily be expected. The PRiMA knockout mouse is an excellent model for cholinergic hyperfunction, because (a.) residual AChE activity is less than 10% of that in WT mice [11]; (b.) striatal ACh levels in these mice were reportedly very high and (c.) these mice are phenotypically normal and seizure-free with only minor problems of motoric function [12]. In the present study, we corroborate our previous report of strongly increased ACh levels in striatum, and we also demonstrate increased levels in hippocampus. Next, we show that the residual AChE activity is still controlling ACh levels to a large extent, because infusion or injection of AChE inhibitors was able to further increase ACh levels several-fold. In this situation, we decided to investigate the functionality of M_2_ receptors which are known to limit ACh release when ACh levels are high. When protein levels of M_2_ receptors were investigated in previous studies, they were reported to be down-regulated by 40–60%, but not absent, in striatum and hippocampus of PRiMA knockout mice [12,13]. The functional consequences of this down-regulation, however, had not been reported.

Presynaptic muscarinic receptors are GPCR which couple to several signaling pathways to reduce ACh release from cholinergic terminals [21]. In untreated rodents, M_2_ receptors limit ACh release when extracellular ACh levels are high; they are not activated by physiological levels of ACh [22]. When ACh levels are increased by AChE inhibitors, blockade of presynaptic M_2_ receptors leads to a several-fold further increase of ACh release, a finding that was corroborated in numerous microdialysis studies in the past years [3,22,23]. As outlined above, however, chronic increases of ACh levels down-regulate M_2_ receptors, with unknown consequences for the regulation of ACh release. Using wild-type mice, we show in this study that oxotremorine, a muscarinic agonist with M_2_ selectivity *in vivo* [24], reduces basal ACh concentrations in striatum to non-detectable levels when given either locally or systemically. In PRiMA KO mice, however, infusion of oxotremorine did not affect the high levels of ACh in striatum. We reasoned that the lack of effect of oxotremorine in PRiMA KO mice may be due to the high ACh levels in those mice which likely saturate presynaptic M_2_ receptors so that additional oxotremorine would be inactive. Indeed, when we infused neostigmine into WT mice to acutely inhibit AChE and increase ACh levels, additional oxotremorine did not affect ACh levels any more. Therefore, the question whether M_2_ receptors were inactive or simply saturated in PRiMA KO mice had to be answered with an additional experiment.

To complement the oxotremorine data, we used scopolamine, a muscarinic antagonist that blocks all muscarinic receptors including the presynaptic M_2_ receptors. This blockade relieves the neurons of M_2_-mediated feedback inhibition of ACh release. This action is usually not seen under basal conditions when presynaptic M_2_ receptors are not activated. Accordingly, in our study, ACh levels in WT mice did not respond to local or systemic scopolamine. When neostigmine is present, however, ACh levels rise and activate presynaptic M_2_ receptors [3,22]. In this situation, scopolamine, either infused locally or given systemically, blocked the negative feedback signal and caused a several-fold increase of ACh release in WT mice (Fig 3). Importantly, extracellular concentrations of ACh in PRiMA KO mice, although high, were not influenced by scopolamine administration. This finding conclusively demonstrates that presynaptic M2 receptors in PRiMA KO mice, which have continuously high ACh levels, have become inactive. Farar *et al*. [12] reported a decrease of 46% and 65% for M_1_ and M_2_ mAChRs, respectively, in the striatum of the PRiMA KO mouse. Our findings here show that the M_2_-mediated effects on ACh release are basically absent in the PRiMA knockout mice. This suggests that the number of functional mAChRs (M_2_) located at the membrane is probably much lower than that originally determined in radiolabeling studies. A possible explanation for this discrepancy is that radiolabeling studies in homogenates or tissue slices may also detect internalised receptors which are not functional. Alternatively, loss of functionality may also be due to a loss of receptor-effector coupling. Unfortunately, our methodological approach cannot distinguish between these possibilities. It should be noted, however, that these results are compatible with findings regarding the neuromuscular junction in PRiMA KO mice [25].

The (basically complete) inactivation of presynaptic M_2_ receptors raises questions as to the meaning of these results for the human situation. First, an adaptation of cholinergic systems would also be expected during chronic therapy with AChE inhibitors. Here, it should be noted that the extent of AChE inhibition in human subjects on AChE inhibitor therapy is probably below 50% [26], and a down-regulation of receptors may not be present when ACh levels rise only two-fold [16]. Interference of PRiMA-AChE binding has been suggested as a possible means to further decrease AChE activity in the brain, with the advantage that peripheral effects of AChE inhibition would be much less severe than with direct AChE inhibitors [12,27]. In such a situation, down-regulation of central M_2_ receptors would likely occur; in other words, a significant feedback inhibition would only be expected at the beginning of treatment, but it would probably subside during chronic treatment with AChE inhibitors. Would this severe inactivation of muscarinic receptors have negative consequences for the therapeutic use of such compounds, i.e. for postsynaptic responses? In previous work, it was reported that PRiMA KO mice performed normally on most motoric tests, and only subtle differences were found in endurance and gait walk [12]. In the present paper, we tested PRiMA KO mice in three cognitive tests and found no impairments, only a somewhat increased activity (it should be noted that improvements in these tests in young and healthy mice are not usually detectable with AChE inhibitors). Our findings are compatible with the idea that, while presynaptic feedback inhibition disappears, postsynaptic neurotransmission which occurs via M_1_ receptors is unchanged or at least preserved under conditions of high ACh levels, but further work is required to corroborate this speculation.

## Supporting Information

S1 File Raw data of Figs 1–4. (XLSX)
